# Gel-immersion cholangioscopy-guided laser lithotripsy for difficult common bile duct stones

**DOI:** 10.1055/a-2919-1345

**Published:** 2026-08-11

**Authors:** Tadahisa Inoue, Shun Futagami, Masato Yano, Takuya Doi, Rena Kitano, Kiyoaki Ito

**Affiliations:** 1 Department of Gastroenterology 12703 Aichi Medical UniversityNagakuteAichi PrefectureJapan

Peroral cholangioscopy (POC)-guided lithotripsy is useful for difficult bile duct stones, but visualization can rapidly deteriorate during lithotripsy because of fragmented stones, sludge, and turbid bile. Repeated saline irrigation may restore visibility but can increase the intraductal pressure and the risk of cholangitis. Gel-immersion electrohydraulic lithotripsy has recently been reported to maintain cholangioscopic visualization by preventing obscuration from stone fragments with smaller-volume injections. 1​ ​2​ However, reports on gel-immersion laser lithotripsy (LL) remain limited, and its feasibility remains uncertain because gel may theoretically attenuate laser energy compared with saline.

**Fig. 1:**
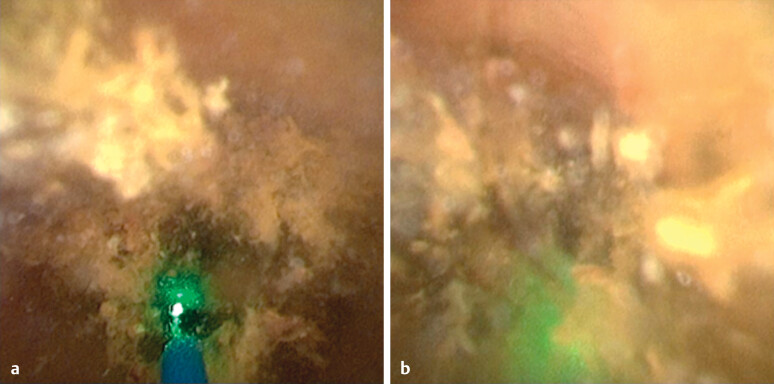
Under saline irrigation, laser irradiation caused rapid dispersion of fine stone fragments and sludge, making the identification of the stone surface difficult ( **a** ). Repeated saline irrigation only temporarily restored visualization, resulting in an unstable cholangioscopic view ( **b** ).

A 74-year-old man with large bile duct stones underwent POC-guided lithotripsy. After endoscopic papillary large-balloon dilation, a cholangioscope was inserted into the bile duct, and a laser fiber was advanced through its working channel. Under saline irrigation, laser irradiation caused rapid dispersion of fine stone fragments and sludge, making the identification of the stone surface difficult ( Fig. 1 ). Repeated saline irrigation only temporarily restored the view, making the procedure time-consuming and inefficient; therefore, we switched to gel immersion with a transparent gel (VISCOCLEAR; Otsuka Pharmaceutical Factory, Tokushima, Japan).

**Fig. 2:**
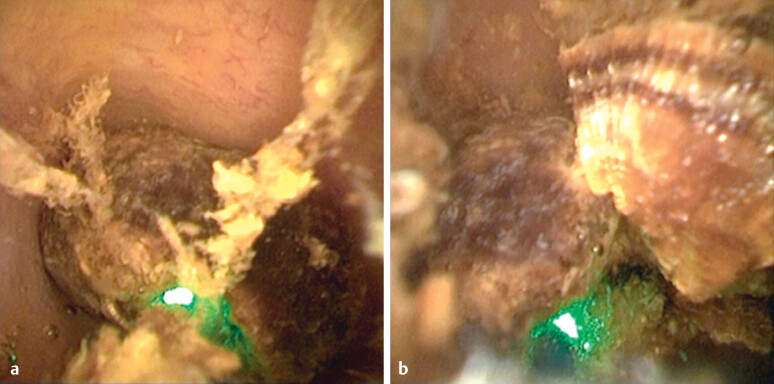
Intermittent injection of approximately 1 mL of gel displaced stone fragments and sludge away from the field of view, restoring clear visualization and enabling continued laser lithotripsy ( **a** and **b** ).

A small amount of transparent gel was injected through the cholangioscope working channel to fill the space in front of and around the stone. Laser irradiation was performed with the fiber tip in contact with or close to the stone surface. Stone fragmentation was achieved without apparent reduction in lithotripsy efficiency. When the visual field began to deteriorate, intermittent injection of approximately 1 mL of gel displaced stone fragments and sludge away from the field of view, allowing the clear visualization and efficient continuation of lithotripsy ( Fig. 2 , Video 1 ). After sufficient fragmentation, the stones and gel were removed using a balloon and basket catheters. No adverse events, including cholangitis or bile duct injury, occurred.

**Video 1** Gel-immersion cholangioscopy-guided laser lithotripsy for large common bile duct stones.


Gel-immersion POC-guided LL was feasible and may help maintain visualization during lithotripsy.

Endoscopy_UCTN_Code_TTT_1AR_2AL
